# Mesh-less laparoscopic treatment of apical prolapse

**DOI:** 10.52054/FVVO.13.2.013

**Published:** 2021-06-28

**Authors:** A Aleksandrov, A.V. Smith, B Rabischong, R Botchorishvili

**Affiliations:** Department of Gynecological Surgery, University Hospital Estaing, Clermont- Ferrand, France; Department of Obstetrics and Gynecology, Specialised Hospital for Obstetrics and Gynecology SBAGAL Pr. Dimitar Stamatov Varna, Medical University Varna, Bulgaria; Department of Obstetrics and Gynecology, Higueras Hospital, Conception, Chile

**Keywords:** Prolapse surgery, sacrocolpopexy, pectopexy, treatment without mesh

## Abstract

The pelvic organ prolapse (POP) is a common gynaecological problem, affecting nearly 50% of women over 40. The sacrocolpopexy using a synthetic mesh is now considered the “gold standard” for management of women with apical prolapse. In April 2019 the FDA placed a ban on the production of transvaginal meshes for prolapse due to late complications. The meshes for abdominal repair of POP are still used, but in future they may also be prohibited. The goal of the following video is to present a mesh-less modification of two techniques used for apical organ prolapse, the sacrocolpopexy and the pectopexy.

## Introduction

The pelvic organ prolapse (POP) is a condition affecting nearly 50% of women over 40 (Abhyankar et al., 2019). The sacrocolpopexy has become the procedure of choice and nowadays is considered the “gold standard” for the management of women with apical prolapse (Barber and Maher, 2013). Numerous studies have demonstrated the advantage of this procedure using a polypropylene mesh over the other types of meshes or the transvaginal repair techniques, mainly due to the significantly lower rates of recurrence (Brubaker et al., 2010; Maher et al., 2016). The main risks of the sacrocolpopexy are the mesh-related complications such as vaginal erosion, dyspareunia, infection and spondylodiscitis (Müller et al., 2020). The pectopexy has been developed in recent years as a technique suitable for patients with difficult dissection of the promontory (Banerjee and Noé, 2011). In April 2019 the FDA placed a ban on the production of all types of transvaginal meshes for prolapse due to complications such as erosion, vaginal bleeding, pelvic pain and dyspareunia. In the near future, meshes used in the abdominal repair of POP could also fall under these restrictions and gynaecologists will be forced to find an alternative management option for these patients (Veit-Rubin et al., 2019). The goal of the following video is to present a modification of the two techniques used for apical organ prolapse, the sacrocolpopexy and the pectopexy, without using a synthetic mesh (https:// vimeo.com/497559243/eb7a724607).

## Surgical technique

The patient is placed into the lithotomy position. Low abdominal pressures of 8-10 mmHg with humidified CO2 is used to decrease the postoperative pain and to reduce peritoneal inflammation. (Matsuzaki et al., 2017). Trocars are placed in standard positions, with one optical trocar in the umbilicus and three 5 mm trocars placed in the suprapubic area. An informed patient consent was obtained from all the patients to use the recorded videos from their surgeries for scientific purposes.

## Sacrocolpopexy

The procedures starts following the steps of the standard sacrocolposuspension (Acsinte et al., 2018). In contrast to the procedure with mesh, extensive dissection of the pararectal space along the uterosacral ligament is not needed, as there is no prothesis to be covered. A dissection of the rectovaginal space then follows, reaching the level of the puborectalis muscles. Next, the surgeon dissects the vesicovaginal space, with the depth of the dissection depending on the extent of the cystocoele. The surgeon then performs a subtotal hysterectomy. After dissecting the spaces, the surgeon performs a posterior colporrhaphy using nonabsorbable polypropylene suture Surgipro size 2/0 (Covidien, Mansfield, MA, USA). The suture starts from the deepest point of the rectovaginal dissection. The surgeon passes the needle transversally from the left to right side of the vagina, ascending towards the cervical stump. Three to four courses are usually sufficient to reach the uterosacral ligaments, where the final stitch of the colporrhaphy is done.

Using same type of suture, the surgeon starts the anterior colporrhaphy from the deepest point of the vesicovaginal dissection. Multiple transversal sutures are made, ascending toward the cervix, where they pass deep in order to secure the suspension.

The surgeon starts the promontofixation from the cervical stump, using a non-absorbable polypropylene suture Surgipro size 0 (Covidien, Mansfield, MA, USA). The surgeon passes the suture along the projection of the uterosacral ligament towards the promontory. Reaching the level of the promontory, the surgeon makes a single left-hand stitch in the anterior longitudinal ligament. The stitch should be approximately 1 cm in length, to ensure solidity, and not to penetrate too deep in order to avoid damaging the intervertebral disc.

After suspension to the promontorium, the needle is driven along the uterosacral ligament in the opposite direction towards the cervix. The suture is tightened using extracorporeal knots, with caution not to generate excessive tension. Peritonisation is done to completely cover the suspension to prevent bowel complications.

## Pectopexy

The surgeon starts the dissection in the crossing point between the umbilical artery and the round ligament to enter the paravesical fossa. The dissection leads directly to the pubic bone and Coopers ligament. Care should be taken not to lacerate the “corona mortis” - an anastomosis between the external iliac and the obturator veins. The dissection is performed on both sides. A subtotal hysterectomy then follows, with attention to spare the round ligaments by cutting them at the level of the uterine cornua, as they are required for the suspension.

The surgeon makes a perpendicular stitch using a nonabsorbable suture Ethibond size 1 (Ethicon, Somerville, New Jersey, USA) into the Coopers ligament, lateral to “corona mortis”. The location of the suture is important, as in the upright position it provides more physiological suspension of the cervical stump. The surgeon drives the suture along the round ligament, which was spared during the hysteresctomy and passes the needle deep through the stump to provide stability. The surgeon then drives the suture backwards along the round ligament, until its entry point is reached on the pelvic sidewall.

The same steps are performed on the left side. A balanced suspension of both sides of the stump should be made. High degree of asymmetry needs to be revised in order to ensure equal traction and to prevent postoperative pain. A subsequent peritonisation using Monocryl size 0 (Ethicon, Somerville, New Jersey, USA) is performed to cover the round ligaments and the cervical stump.

## Discussion

The necessity for alternative techniques replacing the existing methods using synthetic meshes in gynaecological surgery is an idea that emerged in recent years and mainly affects the fields of urogynaecology and prolapse surgery (Veit- Rubin et al. 2019). Krause described a technique for placing a laparoscopic suture the along the uterosacral ligament, suspending the cervix to the sacral promontory with preservation of the uterus (Krause et al., 2006). In our department, a subtotal hysterectomy is the standard technique when performing sacrocolpopexy or pectopexy, with or without a mesh, as it has been shown in our experience to lead to excellent postoperative results, high level of patient satisfaction and low recurrence rate. As it has been shown in the study by Krause, performing a hysteropexy with uterus preservation could also be a valuable alternative. Seracchioli and his team have described their techniques for suspension of the cervix to the sacral promontory along with a subtotal hysterectomy, emphasising the “mesh-less” management of POP (Seracchioli et al., 2018; Paolo et al., 2020). The data from their studies highlight promising results, with a 6.5% recurrence on median follow-up of 24 months. The authors report 10.9% “de novo” constipation with their method using only a suture for the suspension compared to the 10% to 50% constipation found in patients with conventional sacrocolpopexy using mesh (Maher et al., 2011).

To date, no authors have described a modification of the pectopexy using a suture instead of mesh. The sacrocolpopexy has been proven as the gold standard for multi-compartment prolapse and it is the procedure of choice in our department. The pectopexy is chosen over the sacrocolpopexy in obese patients and in patients with difficult access to the promontory (distended sigmoid colon, low aortic bifurcation). Usually, we do not compare the axes of suspension in sacrocolpopexy and pectopexy, but according to studies both techniques have similar postoperative results (Biler et al., 2018; Noé et al., 2015). Guenther Noé demonstrated a laparoscopic technique for anterior and posterior repair of midline defects on the vaginal fascia using native tissues (G. K. Noé et al., 2019). The author applied the technique in combination with other procedures for apical suspension (sacrocolpopexy or pectopexy) and demonstrated excellent results in terms of recurrence and low complications rate. Nonabsorbable polypropylene sutures were selected for the colporrhaphies and for the suspension in our video as it provides more strength to the suspension. The risk of exposure is decreased by using a monofilament suture and by avoiding the complete passage of the needle through the vaginal wall during suturing. So far there is no consensus what type of suture material should be used for the colporrhaphy, as it is clear that a nonabsorbable suture for the apical suspension leads to better results (Bergman et al., 2016; Zebede et al., 2013). Absorbable sutures can be used for the colporrhaphy, as been highlighted by Noé with the slow absorbable type being associated with less risk of symptomatic recurrence (Noé et al., 2019; Bergman et al., 2016).

The modifications of the conventional surgical techniques, that we demonstrate in our video, a pectopexy and a sacrocolpopexy with native tissues fascia repair, are inspired by potential need of the gynaecologist to search for an alternative way to manage patients with POP without using a synthetic prothesis. With our video we present a feasible and reproducible approach using only sutures for the suspension of the cervix and the vagina. Advanced skills in laparoscopic suturing and excellent knowledge of the pelvic anatomy are the prerequisites to perform the corresponding techniques. The data from studies examining these new concept mesh-less techniques show promising short-term results with low complication rates compared to the procedures using prothesis. Further studies are needed to evaluate and determine the optimal way of management of patients with apical POP using a mesh-less approach.

## References

[1] Abhyankar P, Uny I, Semple K (2019). Women’s experiences of receiving care for pelvic organ prolapse: a qualitative study.. BMC Womens Health.

[2] Acsinte O, Rabischong B, Bourdel N (2018). Laparoscopic Promontofixation in 10 Steps.. J Minim Invasive Gynecol.

[3] Banerjee C, Noé K (2010). Laparoscopic pectopexy: a new technique of prolapse surgery for obese patients.. Arch Gynecol Obstet.

[4] Barber M, Maher C (2013). Epidemiology and outcome assessment of pelvic organ prolapse.. Int Urogynecol J.

[5] Bergman I, Söderberg M, Kjaeldgaard A (2016). Int Urogynecol J. Does the choice of suture material matter in anterior and posterior colporrhaphy.

[6] Biler A, Ertas I, Tosun G (2018). Perioperative complications and short-term outcomes of abdominal sacrocolpopexy, laparoscopic sacrocolpopexy, and laparoscopic pectopexy for apical prolapse.. International Braz J urol.

[7] Brubaker L, Maher C, Jacquetin B (2010). Surgery for Pelvic Organ Prolapse.. Female Pelvic Med Reconstr Surg.

[8] Krause H, Goh J, Sloane K (2005). Laparoscopic sacral suture hysteropexy for uterine prolapse.. Int Urogynecol J.

[9] Maher C, Feiner B, Baessler K (2016). Surgery for women with apical vaginal prolapse.. Cochrane Database Syst Rev.

[10] Maher C, Feiner B, Baessler K (2011). Surgical management of pelvic organ prolapse in women: the updated summary version Cochrane review.. Int Urogynecol J.

[11] Matsuzaki S, Vernis L, Bonnin M (2017). Effects of low intraperitoneal pressure and a warmed, humidified carbon dioxide gas in laparoscopic surgery: a randomized clinical trial.. Sci Rep.

[12] Müller P, Berchtold C, Kuemmerli C (2020). Spondylodiscitis after minimally invasive recto- and colpo-sacropexy: Report of a case and systematic review of the literature.. J Minim Access Surg.

[13] Noé G, Alkatout I, Schiermeier S (2018). Laparoscopic anterior and posterior native tissue repair: a new pelvic floor approach.. Minim Invasive Ther Allied Technol.

